# Calorie Compensation Patterns Observed in App-Based Food Diaries

**DOI:** 10.3390/nu15184007

**Published:** 2023-09-16

**Authors:** Amruta Pai, Ashutosh Sabharwal

**Affiliations:** Department of Electrical and Computer Engineering, Rice University, Houston, TX 77005, USA; ashu@rice.edu

**Keywords:** MyFitnessPal, calorie compensation, food diary, preload, mhealth, statistical analysis, nutrition

## Abstract

Self-regulation of food intake is necessary for maintaining a healthy body weight. One of the characteristics of self-regulation is calorie compensation. Calorie compensation refers to adjusting the current meal’s energy content based on the energy content of the previous meal(s). Preload test studies measure a single instance of compensation in a controlled setting. The measurement of calorie compensation in free-living conditions has largely remained unexplored. This paper proposes a methodology that leverages extensive app-based observational food diary data to measure an individual’s calorie compensation profile in free-living conditions. Instead of a single compensation index followed in preload–test studies, we present the compensation profile as a distribution of days a user exhibits under-compensation, overcompensation, non-compensation, and precise compensation. We applied our methodology to the public food diary data of 1622 MyFitnessPal users. We empirically established that four weeks of food diaries were sufficient to characterize a user’s compensation profile accurately. We observed that meal compensation was more likely than day compensation. Dinner compensation had a higher likelihood than lunch compensation. Precise compensation was the least likely. Users were more likely to overcompensate for missing calories than for additional calories. The consequences of poor compensatory behavior were reflected in their adherence to their daily calorie goal. Our methodology could be applied to food diaries to discover behavioral phenotypes of poor compensatory behavior toward forming an early behavioral marker for weight gain.

## 1. Introduction

Uncontrolled weight gain leads to obesity, increasing the risk of cardiovascular diseases and diabetes [1]. Food regulation is essential to maintaining a healthy weight [2]. One of the critical behaviors in food regulation is the compensation of energy content (calories), i.e., adjusting the current meal’s energy content based on the energy content of the previous meal(s) [2,3]. A light dinner salad after a heavy brunch is an example of meal compensation. Compensation may also occur across days. An example of day compensation is eating lower calories after overindulging on a cheat day. Poor compensatory behaviors could be an early behavioral marker for weight gain. However, there is scant research on the methodologies that characterize compensatory behaviors in free-living conditions.

Past research studying compensatory behaviors in adults predominantly adopted a preload–test design [2,3,4,5,6]. Preloads with fixed food attributes are provided to participants. Their effect on a subsequent eating occasion called the test meal is measured in a controlled environment, e.g., in a laboratory. However, preload–test studies provide a limited view of assessing compensatory behavior restricted to controlled laboratory conditions. First, the preload and test meals may not be items the participants consume in their habitual diet. Second, the studies determine compensation only at the next meal. However, compensation may occur later in the day or across days in free-living conditions. There are two opportunities for meal compensation on a given day, i.e., lunch and dinner. Since food habits significantly differ across meal occasions, one may expect different compensation patterns at lunch and dinner.

In this paper, we take a different approach to characterizing the compensatory behaviors of adults in free-living conditions. We leveraged a large public food diary dataset from users of the MyFitnessPal app [7] to apply our proposed methodology. We make the following contributions to further the knowledge of compensatory behaviors exhibited by individuals in real-life conditions.

A computational measure of compensatory behavior: We developed a methodology to determine compensatory behavior at lunch, dinner, and across days. Thus, the methodology allows us to study meal and day compensation. Our methodology characterizes the change in calories at a meal occasion relative to the change in calories at preceding meal/s to assess meal compensation. In contrast, the change in total calories consumed on a day relative to the change in total calories on the preceding day is examined for day compensation. In both cases, change is measured as a deviation from the median calories consumed in the past seven days, assuming that resting metabolic energy expenditure and routine activities would be similar in the past seven days. The preceding meal or preceding day is collectively referred to as a free-living preload. The current meal or day under consideration is collectively referred to as a free-living test. Our methodology classifies each instance of meal or day compensation into one of the four classes: *precise compensation*, *under-compensation*, *overcompensation*, *and non-compensation*. A compensation profile is constructed for each person that summarizes their likelihood for each class of compensatory behavior. By comparing compensation profiles estimated over varying lengths of food diaries, we empirically observed that four weeks of food diary records were sufficient to build an accurate compensation profile of the individual.

Patterns of compensatory behavior in MyFitnessPal users from free-living food diary records: Using the proposed methodology, we computed compensation profiles of 1622 users in the MyFitnessPal food diary dataset. We observed that dinner compensation was more likely than lunch compensation. We found that 90% of users compensated at dinner, and 72% compensated at lunch for more than 50% of the days. There was significant evidence of day compensation, such that 41% of users compensated across consecutive days for more than 50% of the days. Diving deeper into the classes of compensatory behavior, we found that precise compensation was extremely rare. The average likelihood for precise compensation was 6% for lunch, 10% for dinner, and 6% for day compensation. People tended to undercompensate at dinner and overcompensate at lunch. We investigated whether compensation was more for missing calories than additional calories. We found that overcompensation was significantly more likely for negative changes than positive changes in preceding intake. It was 4% more likely at lunch, 4% at dinner, and 5% across days. Not compensating after an increase preceding meal intake and overcompensation after a decrease in preceding calorie intake are poor compensatory behaviors that may lead to a high overall calorie intake. We observed that 41% of users did not meet their calorie goal on the days when they did not compensate at dinner for the increase in lunch and breakfast intake. We found that 34% of users did not meet their calorie goals on the days when they overcompensated at dinner for decreases in lunch and breakfast intake.

With the growing acceptability of smartphone diet monitoring apps, food diary information for a long duration and from a large sample size can be gathered [8,9]. Publicly available food diary datasets, such as the MyFitnessPal dataset [7], allow a unique opportunity to study compensatory behavior in hundreds of free-living individuals with food diaries spanning across weeks. However, food diary data generated via diet monitoring apps remains untapped for building insights into eating behavior patterns. The behavioral patterns discovered in the analyzed dataset give us clear insights into the state of compensation exhibited by diet logging app users. Quantifying their compensation profile with four weeks of diet logs may spark the design of recommendations or just-in-time interventions to modify their compensation profile for higher adherence to their calorie goals.

## 2. Related Work

Calorie Compensation Studies: Calorie compensatory behavior has been traditionally studied with a preload–test study design. The test meal is often provided to all participants after a fixed time period after the preload meal. The preloads are designed to evaluate the impact of different energy content, densities, and macronutrient compositions on the test meal intake [2,4]. Participants of varying age, gender, and BMI are considered to understand the impact of these variables on energy compensation [2,5,6]. Such preload–test studies found that adults often failed to adjust food intake accurately to preloads of various energy densities. The compensation ability declined with age [5]. The findings also suggest that individuals compensate more easily for missing energy instead of the additional energy provided in the preload [5]. However, preload–test studies assess only a single instance of compensatory behavior in a controlled setting. An observational study [10] inspected short-term compensatory behaviors in free-living conditions over 7-days with 24-h recall food diaries. The study found a negative correlation between energy intake on consecutive eating occasions, suggesting the presence of substantial meal compensation in free-living conditions. The compensation was, however, imprecise, leading to considerable variation in total daily intake across the seven days. The study did not investigate whether the imprecise compensation was due to under-compensatory or over-compensatory behaviors. It also did not investigate compensation across days. Additionally, the study duration was limited to 7 days.

Analysis of App-based Food Diaries: Computational studies of app-based food diaries have focused on the recommendation of food substitutes [11] or predicting next day food items [12]. A few recent studies used app-based food diary datasets to assess population-level dietary behaviors, such as consumption of different food groups (e.g., fruits and vegetables) [13,14] or habitual consumption [15]. Other works have identified clusters of users with similar dietary behaviors using text analysis methods on food diaries [16,17]. While previous studies have shed light on certain aspects of dietary behavior, to our knowledge, there has been no study of calorie compensation behaviors using app-based food diary data.

## 3. Materials and Method

Our objective was to extract patterns of compensation from the calorie intake sequence constructed from longitudinal app-based food diaries. Hence, the input “signal of interest” for our method is the individuals’ calorie intake at different meal occasions. We used a public self-reported food diary dataset of MyFitnessPal [7] users to study their compensation patterns.

### 3.1. Preprocessing MyFitnessPal Food Diary Dataset

We used the MyFitnessPal food diary dataset created by Weber and Achananuparp [7,14]. The dataset contains 587,187 days of food diary entries collected from 9896 individuals spanning six months from September 2014 to April 2015. The participating individuals belonged to an online weight loss community and used MyFitnessPal to log their food. The dataset contains demographic information on gender and age group for most users. Female users comprise 73% of the dataset, and male users comprise 16%. The age group from 18 to 44 years comprise 71% of the dataset and greater than 44 years comprise 18%.

A typical food diary entry of a day consists of textual and nutritional descriptions of the meals consumed. The descriptions contain the following information: (i) de-identified participant number, (ii) date of a food log, (iii) the meal occasion label input by the user or default set by the MyFitnessPal application (e.g., breakfast, lunch), (iv) the names of dishes consumed in the meal, (v) nutrition of each dish (e.g., calories, protein, fat), and (vi) MyFitnessPal app provided calorie goal for the day. The calorie intake is a signal of interest in this work. Therefore, we preprocessed the data to analyze each user’s calorie information in the kcal units.

The default labels in MyFitnessPal for meal occasions are breakfast, lunch, dinner, and snacks. However, the app allows users to customize the labels. As a result, some users changed their labels, leading to nonuniform labels in the dataset. To enable user comparison, we excluded individuals with non-default meal occasion labels.

We excluded days that consisted of dishes with calorie information that was negative or more than 3000 calories, as it could be a result of erroneous logging [12]. Self-reported data commonly suffers from missing information in the dataset due to irregular logging. We included users who were complete and consistent in their food records. We excluded days that did not have an entry for each meal occasion (breakfast, dinner, lunch, and snacks). Furthermore, individuals that had recorded complete days consistently were included, i.e., the average number of missing days between the three complete days (less than half a week).

For the purposes of our analysis, we set lunch intake to be a combination of logged lunch and snack calories since we did not have the time information on the consumption of the snacks. After preprocessing the dataset, we conducted an analysis on 4778 users.

### 3.2. Computational Measure of Compensatory Behavior

#### 3.2.1. Notations

The key information of interest is an individual’s daily calorie intake sequence at each meal occasion, namely breakfast, lunch, and dinner. Midday intake is the combination of breakfast and lunch intake. Total intake is the combination of breakfast, lunch, and dinner intake. Let the label for breakfast be b, lunch be l, dinner be d, midday be m, and day be y. The calorie intake sequence of a single user is denoted as $k_{o}\left[n\right]$ where $o\in \left\{b,l,d,m,y\right\}$ is the meal occasion. The variable n refers to the $n^{\;}$ day in the food records of the user. The midday intake for day $n^{\;}$ is $k_{m}\left[n\right]=k_{b}\left[n\right]+k_{l}\left[n\right]$. The total intake for day *n* is given as $k_{y}\left[n\right]=k_{b}\left[n\right]+k_{l}\left[n\right]+k_{d}\left[n\right]$. The median of past 7 days of calorie intake is given as median ${\left(k_{o}\left[i\right]\right)}_{i=n-7}^{i=n-1}$. The deviation from usual intake on a given day n is denoted as $\Delta_{o}\left[n\right]=k_{o}\left[n\right]-\mathrm{median}{\left(k_{o}\left[i\right]\right)}_{i=n-7}^{i=n-1}$. The daily goal is denoted as $g\left[n\right]$, and the daily adherence is denoted as $a\left[n\right]=g\left[n\right]-k_{y}\left[n\right]$, which is the difference between the goal and total calories.

#### 3.2.2. Definitions

In this section, we introduce key terms computed from the calorie intake sequences to quantify compensatory behavior. Previous literature defined calorie compensation as the adjustment of the current meal’s energy content based on the energy content of previous meal(s) [2,3]. The previous meal(s) is referred to as a preload, and the current intake is referred to as a test. Past works [2,3,4,5,6] measured compensation with a controlled preload–test study design. A pair of preload meals are specifically designed for contrast, for example, low energy density vs. high energy density meals. The test meal intake after each preload meal is measured. The difference in test intakes is compared with the difference in preload intakes to compute compensatory behaviors [2,3,4,5,6]. In this work, we computed compensation behavior across meals and days to capture both free-living meal-to-meal and day-to-day fluctuations in calorie intake.

Compensation: Compensation may occur across meals and across days. We defined meal compensation as the change in intake at a meal occasion relative to the change in intake at preceding meal occasion(s). Compensation may occur at lunch and dinner. Compensation at lunch is a change in intake at lunch relative to a change in intake at breakfast. Compensation at dinner is the change in intake at dinner relative to the change in combined breakfast and lunch intake. We defined day compensation as the adjustment in overall intake on a day relative to the change in intake on the preceding day.

In the dataset, there are no preload meals or test meals. The free-living meals are not controlled, blinded, or consistent across participants. However, inspired by the conceptual framework of past works, we introduce the concept of a free-living preload–test pair and present a new approach to calculating compensation patterns from the free-living preload–test pairs.

Free-living preload and free-living test: For calculation of meal compensation, the free-living preload–test pair needs to be meals. Thus, calories consumed in any meal is a free-living test meal intake, and the calorie intake consumed before that meal is the free-living preload meal intake. For calculation of day compensation, the total calorie intake of any day is the free living test day intake, and the total calorie intake of the previous day is the free living preload day intake. We denoted preload on day n as $k_{p}\left[n\right]$ and the test on day n as $k_{t}\left[n\right]$. Note that the preload $k_{p}\left[n\right]$ and test $k_{t}\left[n\right]$ intake correspond to meal intakes in meal compensation computation and total daily intake in day compensation computation.

We calculated deviation from the median intake over the past 7 days to quantify the change in preload and change in test intake. Our intuition behind using the median of the past 7 days as a reference is that we are comparing deviations from usual intake for the preload and test.

Change in free-living preload and test: The change in preload on day n is denoted as $\Delta_{p}\left[n\right]$, and the change in test is denoted as $\Delta_{t}\left[n\right]$. The change in preload $\Delta_{p}\left[n\right]$ is calculated as $k_{p}\left[n\right]$—median ${\left(k_{p}\left[i\right]\right)}_{i=n-7}^{i=n-1}$. Similarly, the change in test $\Delta_{t}\left[n\right]$ is calculated as $k_{t}\left[n\right]-\mathrm{median}{\left(k_{t}\left[i\right]\right)}_{i=n-7}^{i=n-1}$. We compared the magnitude and direction of change in the test to the change in preload to identify the class of compensatory behavior.

Compensation classes: We introduce four classes of compensation behaviors, namely under-, over-, precise, and non-compensation. Compensation for each preload–test pair is classified into one of the four classes: under-, over-, precise, and non-compensation. Under-compensation occurs when the change in the test meal is in the opposite direction of the change in preload meal, and the magnitude of change in preload is less than the change in test by 20%. Overcompensation occurs when the change in the test is in the opposite direction of the change in preload, and the magnitude of the change in test intake is greater than the change in the preload by 20%. Precise compensation occurs when the change in the test is in the opposite direction of preload, and the magnitude of change in the test and magnitude of change in the preload differs by less than 20%. Finally, if the change in test intake is in the same direction as the change in preload intake, then it is referred to as non-compensation. We chose a threshold of 20% because nutrition labels can be up to 20% inaccurate by FDA regulations.

(1) $$c_{t}\left[n\right]=\left\{\begin{array}{cc} \;\mathrm{under}\; & \;\mathrm{if}\;-0.8<\frac{\Delta_{t}\left[n\right]}{\Delta_{p}\left[n\right]}<0\\ \;\mathrm{over}\; & \;\mathrm{if}\;\frac{\Delta_{t}\left[n\right]}{\Delta_{p}\left[n\right]}<-1.2\\ \;\mathrm{precise}\; & \;\mathrm{if}\;-1.2\leq \frac{\Delta_{t}\left[n\right]}{\Delta_{p}\left[n\right]}\leq -0.8\\ \;\mathrm{not}\; & \;\mathrm{if}\;\frac{\Delta_{t}\left[n\right]}{\Delta_{p}\left[n\right]}>=0 \end{array}\right.$$

The compensation behavior sequence was calculated for each lunch, dinner meal and each day. We collectively denoted the compensation behavior sequence as $c_{t}\left[n\right]$, where t∈ $\left\{l,d,y\right\}$ indicate lunch, dinner, and day compensation, respectively. The elements in the compensation behavior sequence belong to the compensation class set $S=\left\{\mathrm{under},\;\mathrm{over},\;\mathrm{precise},\;\mathrm{not}\right\}$.

For lunch compensation analysis, lunch is treated as a test meal, and breakfast is treated as the preload meal. For dinner compensation, dinner is considered the test meal, and breakfast with lunch together is considered as the preload meal. In day compensation, a test day is any specific day, and the corresponding preload day is the previous day.

Since food logging is a burdensome activity [18], missing days are prevalent in the sequence. While computing day compensation, we only consider the days for which we have complete information for the previous day. Additionally, we ignore days with missing values while calculating the median intake of the past seven days. Our definition requires a non-zero change in preload. We calculated the class of compensatory behavior only for non-zero changes in preload. The number of days when $\Delta_{p}\left[n\right]=0$ was extremely limited in the dataset.

**Figure 1:**
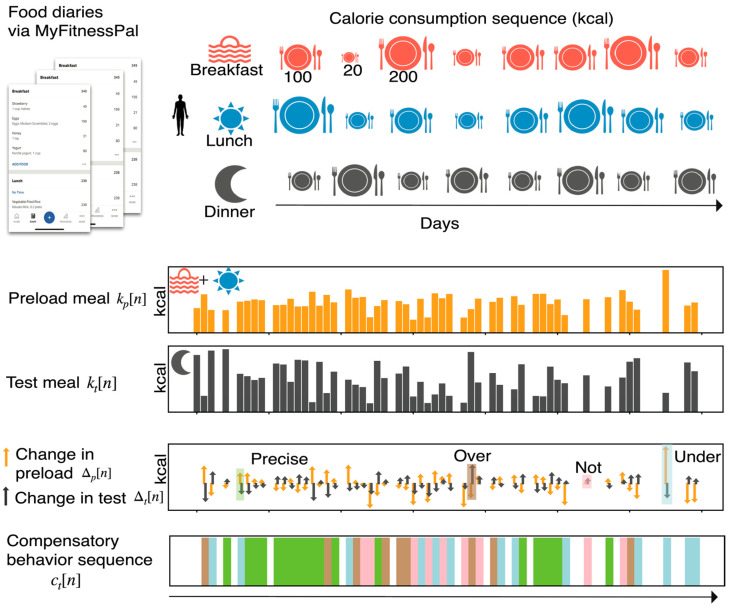
Overall pipeline for the computation of the calorie compensatory behavior sequence. Food diaries of MyFitnessPal users are parsed to construct calorie consumption sequences for breakfast, lunch, and dinner. Computation of compensatory behavior sequence at dinner is shown for an example user. The user’s breakfast and lunch calorie intake sequence is combined as the midday intake sequence. The midday intake is considered as the preload meal, and the dinner intake as the test meal. Compensatory behavior at dinner for each day is identified by comparing changes in test intake to changes in preload, where change refers to deviation from median intake. Based on the relative changes, the day is labeled one of the four classes of compensatory behavior (under, precise, over, not). Finally, a compensatory behavior sequence is constructed, which is then used to summarize the compensation profile of the user.

The pipeline for computation of the calorie compensatory behavior sequence at dinner for an example user is shown in Figure 1. The lunch and breakfast calorie intake is combined and considered as the free-living preload intake. The dinner calorie intake is considered as the free-living test intake. The changes in preload and test are computed for each day. The compensation class for each day is computed with Equation (1). We used the proposed pipeline for each user and constructed the lunch compensation behavior sequence $c_{l}\left[n\right]$, the dinner compensation behavior sequence $c_{d}\left[n\right]$, and the day compensation behavior sequence $c_{y}\left[n\right]$.

#### 3.2.3. Compensation Behavior Measures

Next, we propose computational measures that summarize the behavior sequence and allow comparison among users in the dataset. Comparison among users allows the discovery of population-level trends.

Compensation profile of a user: We computed the compensation profile as the probability distribution function constructed from the samples of calorie compensation behavior sequence. We modeled the calorie compensation sequences $c_{l}\left[n\right],c_{d}\left[n\right],c_{y}\left[n\right]$ as discrete time and discrete space random processes that take one of the following values S={under, over, precise, not} for every day n. From the samples of the calorie compensation behavior sequence, we empirically estimated the probability mass function of the compensation behavior for each user. Probability mass functions (pmf) are denoted as $P_{c_{t}}$, where $c_{t}\in \left\{c_{l},c_{d},c_{y}\right\}$ corresponds to lunch, dinner, and day compensation profiles. The compensation profile of the user represents the fraction of days the user exhibited a class of compensatory behavior.

Based on the direction of change in the preload, the four classes of compensation had varying significance. For example, for the positive change in preload, non-compensation may lead to overall high calorie intake, but for a negative change in preload, non-compensation may signify low daily calorie intake. High calorie intake could result in exceeding the daily calorie goal budget.

Compensation profile conditioned on direction of preload change: We computed the conditional compensation profile for positive $\left(\Delta_{p}\left[n\right]>0\right)$ and negative changes in preload $\left(\Delta_{p}\left[n\right]<0\right)$ separately. Thus, we estimated two conditional probability mass functions (pmf), $P_{c_{t}|\Delta_{p}>0}$ and $P_{c_{t}|\Delta_{p}<0}$, to investigate the differences in the likelihood of compensation classes for positive and negative changes in preload. All conditional compensation profiles were computed for lunch, dinner, and day compensation separately by substituting $\left(c_{t}\left[n\right],\Delta_{p}\left[n\right]\right)$ as each of the tuple elements in $\left\{\left(c_{l}\left[n\right],\Delta_{b}\left[n\right]\right),\left(c_{d}\left[n\right],\Delta_{m}\left[n\right]\right),\left(c_{y}\left[n\right],\Delta_{y}\left[n-1\right]\right)\right\}$, respectively.

Median adherence conditioned on the direction of preload change and compensation class: Adherence measure for each day n denoted as $a\left[n\right]$ quantifies if the user is within or above their designated calorie goal for the day. A positive value for $a\left[n\right]$ implies that the user was within the goal, and a negative value implies that the user exceeded their goal. To understand the impact of each class of compensation given a direction of preload change on the adherence to the calorie goal, we computed the median adherence conditioned on the direction of preload change and compensation class. Given the compensatory behavior sequence $c_{t}\left[n\right]$ and the preload change sequence $\Delta_{p}\left[n\right]$, we computed the median adherence for positive preload changes denoted as ${\tilde{a}}_{c_{t},s,\Delta_{p}>0}$. The median adherence ${\tilde{a}}_{c_{t},s,\Delta_{p}>0}$ is computed as median $\left\{a\left[n\right]|c_{t}\left[n\right]=s,\Delta_{p}>0\right\}$. Similarly, we computed the median adherence for negative preload changes denoted as ${\tilde{a}}_{c_{t},s,\Delta_{p}<0}$. The adherence computation is performed for every compensation class s∈S and each tuple $\left(c_{t}\left[n\right],\Delta_{p}\left[n\right]\right)\in \left\{\left(c_{l}\left[n\right],\Delta_{b}\left[n\right]\right),\left(c_{d}\left[n\right],\Delta_{m}\left[n\right]\right),\left(c_{y}\left[n\right],\Delta_{y}\left[n-1\right]\right)\right\}$ corresponding to lunch, dinner, and day compensation.

## 4. Analysis Results

In this section, we present the analysis of the proposed compensatory behavior measures observed in MyFitnessPal users. We first examined the minimum number of recorded days needed to estimate the compensation profile of the users accurately. Then, we investigated the patterns of the lunch, dinner, and day compensation profiles. Next, we studied the relationship between the direction of preload change and the class of compensatory behavior. Finally, we compared the adherence to calorie goals for different classes of compensation.

### 4.1. Duration for Measurement of Compensatory Behavior

The compensation profiles were constructed from repeated measures data observed over a period of time. The number of days logged varied from user to user. The number of days influenced the accuracy of the estimated profiles. However, we did not know the true underlying behavior distribution to compute its divergences from the estimated profiles. However, we can fairly assume that a greater number of days enables a more accurate representation of the user’s underlying behavioral distribution. Hence, we inspected the dependence between the estimated profiles and the number of days of logs quantitatively. Let N denotes the set of days for which the calorie intake is logged.

We performed the analysis on a subset of users with long consumption sequences. We included a subset of individuals with N≥84 for the analysis of lunch and dinner compensation profiles. There were 426 users with N≥84 for dinner and day compensation computation and 424 users with N≥84 for lunch compensation computation. We used all the days in their sequence to calculate $P_{c_{l}},P_{c_{d}}$, and $P_{c_{y}}$ as the stable compensation profile.

Next, we estimated the compensation profiles with $\hat{N}$ ranging from 14 to 84 days. For each $\hat{N}$, we estimated ${\hat{P}}_{c_{l},\hat{N}},P_{c_{d},\hat{N}}$, and $P_{c_{y},\hat{N}}$ using any random continuous $\hat{N}$ days of the consumption sequence. Next, we computed the distance between the estimated profile and the corresponding stable profile for varying $\hat{N}$ using the Jensen–Shannon Divergence equation [19]. The Jensen–Shannon Divergence between two probability distributions Q,R is denoted as $JSD\left(Q∥R\right)$. It measures the distance between two probability mass functions. The measure has a range of 0 to 1. For each user, we found the average Jensen–Shannon Divergence calculated as $JSD_{c_{t,\hat{N}}}=\sum_{\hat{N}\in N}JSD\left(P_{c_{t},\hat{N}}∥P_{c_{t}}\right)$, where $c_{t}\in c_{l},c_{d},c_{y}$.

**Figure 2:**
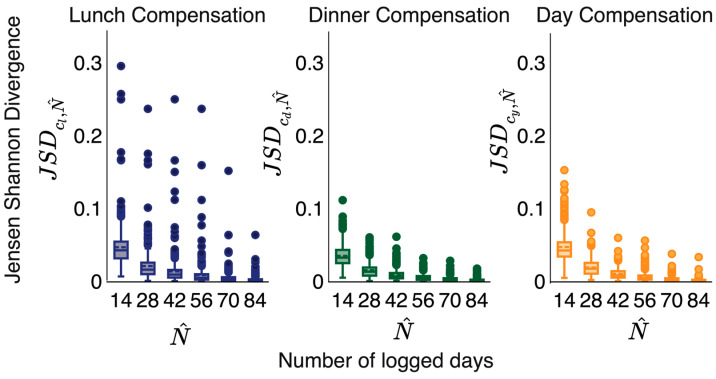
Relationship between the accuracy of the compensation profile and the number of logged days. With the increase in the number of days (sequence length), we achieved a more accurate compensation profile (lower Jensen–Shannon Divergence between estimated compensation profile and stable compensation profile). We observed 28 days to be the elbow of the JSD vs. the number of days curve.

Figure 2 demonstrates that when we include more days in our analysis, our estimated compensation profiles are closer to the stable profiles. We observed the same trend for lunch, dinner, and day compensation. We empirically observed that 28 days appeared as the elbow of the curve using the Kneedle algorithm [20]. As a result, for subsequent analysis, we considered participants with N≥28. There were 1622 users with N≥28 in the dataset.

Food diary records spanning a period of 28 days were sufficient to capture the compensation profile of a user. We considered users whose length of calorie consumption sequence was greater than or equal to 28 to minimize bias in our analysis that may result from insufficient data.

### 4.2. Meal and Day Compensation Profiles

**Figure 3:**
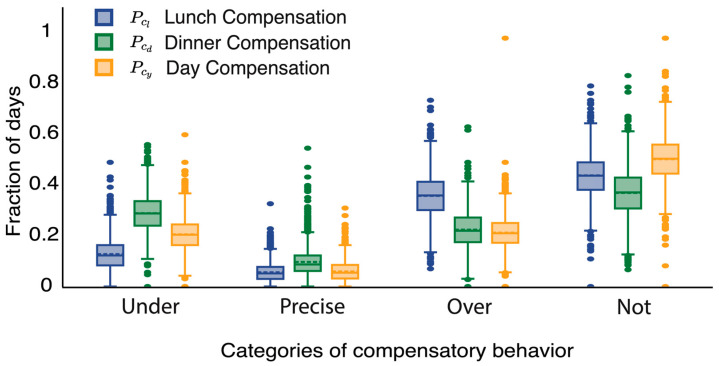
Statistical comparison of compensation profiles of users in the MyFitnessPal dataset for meals and days. The lunch, dinner, and day compensation likelihoods for different categories of compensation are shown in the Figure.

Figure 3 displays a comparison between meal (lunch, dinner) and day compensation across the four different compensation classes. We used the Friedman test [21] for multiple group comparisons and the Wilcoxon signed-rank test [22] for pairwise post hoc comparisons with Bonferroni correction. We observed significant differences in the lunch, dinner, and day compensation profiles of users. The likelihood of non-compensation was highest for the day, followed by lunch and dinner (*p* < 0.001). As a result, users showed compensatory behavior in the opposite direction of change in preload more at the meal level than at the day level.

We found that the likelihood of under-compensation was higher at dinner, followed by day and lunch in order (p<0.001). In contrast, overcompensation was most likely at lunch, followed by dinner and day (p<0.001). We found that the likelihood of precise compensation was very low. The likelihood of precise compensation was the least compared to other classes for meal and day compensation. The mean fraction of days that a user exhibited precise-compensation was $6\%\;\left(\sigma =4\%\right)$ for lunch, $10\%\;\left(\sigma =6\%\right)$ for dinner and 6% $\left(\sigma =4\%\right)$ for day compensation.

Meal-level compensation was significantly more likely than day-level compensation. Compensation at dinner was more likely than lunch. Precise compensation of calories was extremely rare.

### 4.3. Relation between the Category of Compensatory Behavior and Direction Preload Change

**Figure 4:**
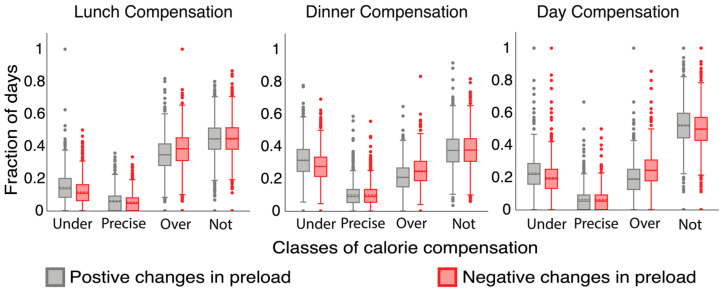
Statistical comparison between compensation profile for positive and negative changes in preload for lunch, dinner, and day compensation levels.

We quantified the differences in compensation profiles for positive and negative changes in preload. Figure 4 shows a statistical comparison between compensation profiles for positive changes in preload $P_{c_{t}|\Delta_{p}>0}$ and negative changes in preload $P_{c_{t}|\Delta_{p}<0}$ for each level of compensation i.e., lunch, dinner, and day. The compensation profiles $P_{c_{t}|\Delta_{p}>0}$ and $P_{c_{t}|\Delta_{p}<0}$ were significantly different for meal and day compensation. Overcompensation was more likely for negative changes than positive changes by $4\%\left[-4\%,11\%\right]$ for lunch compensation (1619 users, p<0.001), by $4\%\left[-3\%,11\%\right]$ for dinner compensation (1624 users, p<0.001) and $5\%\left[-4\%,15\%\right]$ for day compensation (1623 users, p<0.001).

Overcompensation was significantly more likely for negative changes than positive changes in preload at all levels of compensation. Individuals tended to over-adjust for missing calories compared to additional calories.

### 4.4. Relation between the Category of Compensatory Behavior and Adherence to Goal

In our analysis, we computed the median adherence of the individual for each class of compensatory behavior for positive and negative preload changes for lunch, dinner, and day compensation. Table 1 summarizes the percentage of users who had a positive median adherence for the days they exhibited each class of compensatory behavior given direction of change in preload and the level of compensation. Note that a positive median adherence signified that the users were within their goal. For positive changes in preload, the percentage of users with positive median adherence decreases from the overcompensation category to the non-compensation category. In contrast, for negative changes in preload, the overcompensation category had the lowest percentage. The trend supports our conclusion that overcompensation for negative changes in preload leads to the lowest adherence to the calorie goal. For positive changes in preload, non-compensation led to the lowest adherence to calorie goals.

For positive changes in preload, non-compensatory behavior led to the lowest adherence to calorie goals. In the case of negative changes in preload, overcompensation led to the lowest adherence to calorie goal.

## 5. Conclusions

Previous research has predominantly examined compensation behavior through controlled studies conducted in a laboratory. To our knowledge, this is one of the first studies to extensively define and analyze daily compensatory behavior from real-life longitudinal food diary data collected using diet-tracking applications. We developed comprehensive computational measures of compensatory behavior estimated from self-reported food intake records. Our proposed measure distinguishes between meal and day compensation. We conducted a statistical analysis of the compensation profiles of MyFitnessPal users via analyzing a publicly available MyFitnessPal dataset. We investigated how the direction of the change in preload impacts the user’s compensation profile. We also examined the impact of various classes of compensation behavior on adherence to calorie goals. We found population-level patterns that extend the understanding of free-living calorie compensation.

Self-regulation via compensation profile: Poor self-regulation may be a marker of weight gain. A recent study [23] measured self-regulation via calorie compensation indices (COMPX) in college students. We can compute compensation profiles from app-based food diaries to identify gaps in individual-specific self-regulation behaviors. These measures can act as behavioral markers for designing personalized diet management strategies for improving self-regulation.

Compensation across meals or days: The difference in meal and day compensation profiles can be used to find which compensation strategy is attainable for everyone. Meal compensation is necessary for adhering to the total daily calorie quota. Similarly, day compensation may be important to achieve alternate-day modified fasting [24]. Based on specific routines, some individuals might find it easier to compensate at meals, while others may prefer to compensate across days. Among the MyFitnessPal users, we observe a population-level pattern of higher meal compensation than day compensation. There is a higher tendency of compensation at dinner than at lunch for meal compensation. The prominence of meal compensation may be attributed to the daily calorie goal feature of the app, where users aim to stay within their calorie goal every day.

From compensation indices to compensation classes: Contrary to previous studies that measure a single compensation index for each individual, we measure a behavioral distribution across compensation classes. Each class of compensatory behavior has a different implication, conditioned on the direction of change in preload. Overcompensation after a negative change in preload may lead to a positive calorie balance that could accumulate over time if an individual shows frequent overcompensation for negative changes in preload—for example, overeating at lunch after skipping breakfast regularly. However, under-compensation or not-compensation on the days of skipped breakfast could lead to lower energy levels. On the other hand, under-compensation after a positive change in preload could lead to a positive calorie balance—for example, the absence of under-eating after a binge eating episode. Our analysis found that overcompensation behavior is likely after a negative change in preload than a positive change in preload. The finding is similar to prior evidence that individuals adjust more easily for missing than additional energy [5]. We identified poor compensatory behaviors, such as overcompensation for negative preloads or non-compensation for positive changes in preload that lead to low adherence to calorie goals. Reducing the likelihood of such poor compensatory behaviors can help individuals reach their calorie goals more effectively and attain weight goals faster.

A quantitative measure of intuitive eating: Intuitive eating is an adaptive eating strategy defined as eating in response to internal cues of hunger and satiety [25]. Intuitive eating is associated with a lower body mass index [25,26]. We propose that compensation measures capture an individual’s intuitive eating behavior quantitatively. We hypothesize that individuals who exhibit intuitive eating will also show less non-compensatory behaviors. Data on psychometric [27] and quantitative analysis via compensation profiles may help to understand its barriers and effectiveness.

Missing physical activity: A limitation of our analysis is not taking into account physical activity levels between meals. Energy expenditure between meals may influence the compensation of energy intake at meals [28]. For example, non-compensation being followed after a positive preload change could result from energy expenditure through physical activity between preload and test meals. A recent finding states [29] that our body compensates for an increase in levels of physical activity by reducing basal energy expenditure. Hence, imprecise calorie compensation and eating back exercise calories, coupled with the fact that our body internally adjusts for increases in physical activity may affect weight gain adversely. A future direction would be to collect food diary information and timed physical activity expenditures to understand the role of physical activity between meals on compensatory behaviors. Another future step would be to collect datasets that consist of both food diary records and weight measurements over time. Longitudinal analysis to investigate the impact of compensation profiles on weight management goals is needed to establish the significance of compensation profiles as behavior markers for weight gain.

Unblinded calorie information: As opposed to other works on calorie compensation, the preload in our analysis was not blinded. The users of the diet-tracking app had access to the preload’s calorie information. However, we observed significant imprecise compensation and not-compensatory behavior. We acknowledge that our results may be biased due to the calorie information being visible to users. Future study designs may blind the calorie intake and goal information while employing digital diet-tracking tools for capturing the compensatory behavior pattern.

Generalizability: Our dataset consisted of predominately female users and users in the age group between 18 and 44 years. Thus, our findings may have limited generalizability. The individuals in the MyFitnessPal dataset may be more health conscious. However, previous works [13,14] found that individuals who use diet-tracking apps, such as MyFitnessPal, exhibit dietary behaviors comparable to the general population. Additionally, one of the strengths of our study lies in the large sample size of 1622 individuals. Although our analysis was on MyFitnessPal users, the proposed methodology and observed trends could guide repeatability studies in different populations.

In summary, we developed measures to quantify compensatory behavior from free-living food diary records. We then presented an extensive analysis of patterns of compensatory behaviors exhibited by users of the MyFitnessPal diet-tracking application. Future studies should evaluate the role of free-living compensatory behaviors in weight.

## Figures and Tables

**Table 1:** Percentage of users who show a positive median adherence for each category of compensatory behavior given direction of change in preload and the level of compensation.

Percentage of Users (%)						
	Positive Change in Preload			Negative Change in Preload		
	Lunch	Dinner	Day	Lunch	Dinner	Day
	(n = 1149)	(n = 1444)	(n = 1037)	(n = 1047)	(n = 1444)	(n = 1021)
Over	88	89	94	73	66	55
Precise	78	80	88	78	80	66
Under	75	64	87	84	92	75
Not	67	59	62	91	96	93

## Data Availability

The original raw dataset [7] is publicly available at [30]. The custom code developed for this study is available from the corresponding author on reasonable request.
